# Dynamic Weighting Translation Transfer Learning for Imbalanced Medical Image Classification

**DOI:** 10.3390/e26050400

**Published:** 2024-05-01

**Authors:** Chenglin Yu, Hailong Pei

**Affiliations:** 1 School of Electrtronic & Information Engineering and Communication Engineering, Guangzhou City University of Technology, Guangzhou 510800, China; 2 Key Laboratory of Autonomous Systems and Networked Control, Ministry of Education, Unmanned Aerial Vehicle Systems Engineering Technology Research Center of Guangdong, South China University of Technology, Guangzhou 510640, China; 3 Key Laboratory of Autonomous Systems and Networked Control, Ministry of Education, Unmanned Aerial Vehicle Systems Engineering Technology Research Center of Guangdong, School of Automation Scinece and Engineering, South China University of Technology, Guangzhou 510640, China; auhlpei@scut.edu.cn

**Keywords:** imbalanced medical image classification, class distribution entropy, transfer learning, dynamic weighting, cycle translation, confidence-based selection

## Abstract

Medical image diagnosis using deep learning has shown significant promise in clinical medicine. However, it often encounters two major difficulties in real-world applications: (1) domain shift, which invalidates the trained model on new datasets, and (2) class imbalance problems leading to model biases towards majority classes. To address these challenges, this paper proposes a transfer learning solution, named Dynamic Weighting Translation Transfer Learning (DTTL), for imbalanced medical image classification. The approach is grounded in information and entropy theory and comprises three modules: Cross-domain Discriminability Adaptation (CDA), Dynamic Domain Translation (DDT), and Balanced Target Learning (BTL). CDA connects discriminative feature learning between source and target domains using a synthetic discriminability loss and a domain-invariant feature learning loss. The DDT unit develops a dynamic translation process for imbalanced classes between two domains, utilizing a confidence-based selection approach to select the most useful synthesized images to create a pseudo-labeled balanced target domain. Finally, the BTL unit performs supervised learning on the reassembled target set to obtain the final diagnostic model. This paper delves into maximizing the entropy of class distributions, while simultaneously minimizing the cross-entropy between the source and target domains to reduce domain discrepancies. By incorporating entropy concepts into our framework, our method not only significantly enhances medical image classification in practical settings but also innovates the application of entropy and information theory within deep learning and medical image processing realms. Extensive experiments demonstrate that DTTL achieves the best performance compared to existing state-of-the-art methods for imbalanced medical image classification tasks.

## 1. Introduction

The application of deep learning technology has achieved considerable progress in plenty of medical image diagnosis tasks, including diabetic retinopathy grading [1], pathological image classification [2,3,4], and brain MRI images [5,6,7], especially in lesion segmentation [8,9] and diagnosis [10,11]. The supervised convolutional neural network is a widely accepted learning framework in medical situations where sufficient annotated images are provided [12,13,14]. However, the prohibitive cost of professional annotations often renders the ample expert supervision scarce in real clinical scenarios [15,16], causing many challenges for the generalization of AI medical image diagnosis. Moreover, directly adopting pre-trained models from another dataset often results in unreliable performance due to the typical domain shift between different datasets [17,18,19]; for example, the target domain is in a heterogeneous data distribution away from the source on where the model was pre-trained. In particular, the domain shifts in the medical field are usually attributed to variations in imaging protocols, parameters settings, devices, and scanner manufacturers. Consequently, that strongly motivates various attempts [20,21,22] on the unsupervised domain adaptation to promote the diagnostic performance in medical image diagnosis with an available labeled source dataset.

**Figure 1:**
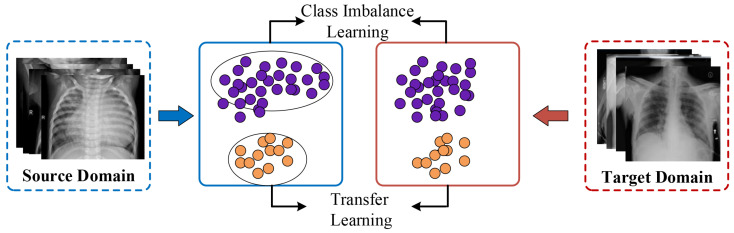
The research task of our unsupervised representation learning framework.

The goal of unsupervised domain adaptation (UDA) is to transform the medical images into a domain-invariant feature space by leveraging a fully labeled source domain. That makes it achievable for applying the source diagnostic model to the unlabeled target domain. The essential point of UDA is to align the domain discrepancy among medical datasets, derived from various imaging settings [20,23]. The representative samples are illustrated in Figure 1. For instances, Fang et al. [20] deployed a discrepancy-based unsupervised domain for cross-domain fMRI patterns of labeled source and unlabeled target samples via an attention-guided graph convolutional module and a maximum mean discrepancy constrained module; Liu et al. [23] adaptively carried out fine-grained subtype-wise compactness with intermediate pseudo-labels to dynamically bridge the domain shift, achieving promising results on a medical diagnosis task.

In addition to the distribution gap that conventional UDA models addressed, medical image classification is usually confused by another two concerns. (a) The imbalanced class distribution in medical diagnosis is more prevalent compared to nature imaging. For example, most skin lesion patients are finally diagnosed with benign lesions, while very few patients are identified with malignancy [24]. When data imbalance occurs, existing DA models tend to correctly identify the major cases and misdiagnose the minor categories. (b) Considerable discrepancies among intra-domain medical images are equally common, such as varying appearance and shapes of tumors, and different imaging steps with tissue sectioning and staining, which produces an inconsistent feature space with challenging discriminability for existing medical domain adaptation (MDA) methods. To better illustrate the goal of this work, we create the original research task in Figure 1 of solving class imbalance learning and transfer learning in unsupervised representation learning.

In summary, the imbalance class distribution and intra-domain inconsistency in medical domain adaptation result in poor diagnostic performance on target medical images even though its cross-domain discrepancy has been bridged properly with source data. Obviously, the diagnosis ability of MDA mainly relied on both the cross-domain alignment and feature discriminability; thus, the above two obstacles must be overcome and deserve significant attention to be drawn to solving them in the theoretical development of medical domain adaptation. Furthermore, former MDA models still do not solve the challenges of imbalanced class and inconsistent distributions between medical domains, giving the domain (class) with larger data numbers greater weight in model optimization. Such problems tend to bias model training, resulting in unsatisfied alignment and severe misdiagnosis. That reflects high-entropy scenarios where certain conditions are over-represented compared to others. This imbalance and variability hinder effective domain adaptation and classification, necessitating approaches that specifically address these entropy-related challenges.

To address these entropy-centric problems that are commonly neglected by previous MDA models [20,21,22], this paper proposes Dynamic Weighting Translation Transfer Learning (DTTL) to align the domain shift for the imbalanced medical image classification task. The designed dynamic transfer learning method can measure each medical image according to their imbalance level among two domains and the proposed balanced translation mechanism generates minority medical images by translating the related majority samples, which extends the decision boundary among the imbalanced classes. In addition, DTTL explores a confidence-based selection method to reserve the most valuable synthesized medical images for further prediction model training. This approach not only addresses the class imbalance but also incorporates principles of entropy reduction by enhancing feature discriminability and extending decision boundaries, thereby reducing the overall entropy in the system.

The major contributions are listed below:

(1) To address the issue of class imbalance in medical image classification, this paper introduces a model based on Dynamic Weighted Transformation Transfer Learning (DTTL) that aligns the cross-domain distribution differences among imbalanced classes, extends the decision boundaries of minority sample categories, and enhances the capability of cross-domain imbalanced class feature learning, thereby reducing entropy and enhancing cross-domain imbalanced class feature learning.

(2) A Dynamic Domain Translation module is proposed, which links discriminative feature learning between the source and target domains through a synthetic discriminability loss and a domain-invariant feature learning loss, achieving cross-domain transfer learning in medical image classification based on entropy-focused mechanisms.

(3) A Balanced Target Learning mechanism is devised, employing a confidence selection method to choose the most useful synthesized images. This method develops a dynamic translation process between the two domains for imbalanced classes to create a pseudo-labeled balanced target domain.

## 2. Related Work

Medical image classification is a challenging task that has received significant attention in recent years. However, two major problems that affect the performance of these models are class imbalance and domain shift. This section aims to summarize related research on these issues.

### 2.1. Class-Imbalanced Medical Image Classification

Class imbalance refers to the situation where the number of images in one class significantly outweighs the others. In medical imaging, some diseases occur less frequently than others, leading to imbalanced medical image datasets. Several approaches have been proposed to handle this problem, including data augmentation, oversampling, and undersampling techniques.

In detail, data augmentation methods, as a conventional solution, have utilized various algorithms such as rotation, flipping, scaling, and cropping to address class imbalance issues in medical image classification tasks. In addition, advanced models propose different strategies for combining data augmentation with other techniques, such as oversampling, undersampling, or weighted loss functions, to improve the performance of deep learning models. For example, Huynh et al. [25] proposed an adaptively blended consistency loss to address the class imbalance problem in semi-supervised medical image classification tasks by adaptively mixing target class distributions, showing improvement in unweighted average recall, making it a promising solution for improving the performance of medical image classification. Liu et al. [26] aimed to solve imbalanced medical image datasets that cause predictions biased towards majority classes. They proposed a semi-supervised deep learning method that leverages uncertainty-guided virtual adversarial training and batch nuclear-norm optimization to improve the discriminability, diversity, and generalization of trained models, achieving better results than state-of-the-art semi-supervised methods on two publicly available datasets and one in-house collected dataset.

Although these existing approaches solve the class imbalance problem in medical image classification, they also require sufficient training data in the same domain. This limitation causes poor performance when the model is employed in different domains.

### 2.2. Transfer Learning-Based Medical Image Classification

Transfer learning-based medical image classification has been an active research area in recent years and the goal is to improve the performance of deep learning models when the distribution of the training data differs from that of the target data, which commonly occurs due to variations in imaging protocols, scanner types, or patient populations.

Several transfer learning techniques have been proposed in medical image classification [21,27], such as adversarial learning, domain confusion, and gradient alignment. These methods adapt the learned features in the source domain to better generalize to the target domain, allowing for improved accuracy and robustness of the deep learning models. Recent studies have shown that domain adaptation can be effective in addressing various problems in medical image classification. In particular, Diao et al. [21] designed a histogram-based generative adversarial network methodology for domain adaptation that outperforms standard pixel-based GAN methods in classifying chest X-rays from various heterogeneous target domains. This is based on the hypothesis that most domain shifts in medical images are variations of global intensity changes that can be captured by transforming histograms along with individual pixel intensities. On the other hand, Mahapatra et al. [27] incorporated graph neural networks and disentangled semantic and domain invariant structural features into an unsupervised domain adaptation framework. This approach yields better medical image classification results across distribution shifts compared to other methods.

In addition, Ganin et al. [28] and Long et al. [29] have both contributed significantly to the field of domain adaptation in neural networks, with the former introducing a method for the domain-adversarial training of neural networks and the latter advancing the field further with conditional adversarial domain adaptation techniques. Then, the landscape of domain adaptation techniques has been enriched by a variety of innovative approaches, such as Cycle Consistent Adversarial Domain Adaptation (CyCADA) [30], which leverages cycle consistency for domain translation, and Beyond Sharing Weights (BSW) [31], which explores structures beyond mere weight sharing for domain adaptation. Additionally, methods like Maximum Classifier Discrepancy (MCD) [32], Margin Disparity Discrepancy (MDD) [33], and FixBi [34] have focused on classifier-based discrepancies to bridge domain gaps. Concurrently, Entropy Minimization versus Diversity Maximization (MEDM) [35] and Contrastive Domain Adaptation with Consistency Match (CDACM) [11] have emphasized the importance of balancing feature alignment with diversity for effective domain adaptation.

The above analyzed transfer learning methods have addressed the negative effects of domain shift in medical datasets. However, these methods often overlook the class imbalance issue, a critical factor that can significantly reduce the applicability and effectiveness of transfer learning in medical image datasets.

## 3. Method

**Problem Definition.** This work tends to solve the unsupervised domain adaptation with imbalanced medical images, which often damage the diagnostic performance in clinical applications. Mathematically, we assume $n_{s}$ the source medical images, $S=\left\{\left(x_{i}^{s},y_{i}^{s}\right)\right\}{|}_{i=1}^{n_{s}}$, with imbalanced majority class $X_{maj}^{s}$ (e.g., benign) and another minority one $X_{min}^{s}$ (e.g., malignancy), and an unlabeled target domain is $T=\left\{x_{j}^{t}\right\}{|}_{j=1}^{n_{t}}$. The target domain does not have any annotation and its class distributions are usually imbalanced according to practical medical scenarios.

The aim of this paper is to develop a successful deep learning diagnostic model for the unlabeled medical domain, integrating both imbalanced labeled source images and inconsistent unlabeled target data. The above task, nevertheless, expresses challenging difficulties from its cross-domain discrepancy and intra-domain imbalanced classes in medical domain. The majority of existing UDA models only bridge the cross-domain discrepancy under balanced class distributions or weigh the transferable images equally to transfer, resulting in unfaithful diagnosis in actual medical scenarios. Therefore, this paper proposes a novel Dynamic Weighting Translation Transfer Learning (DTTL), which intricately weaves the principles of entropy and information theory into its core. This methodology is designed to adeptly tackle the hurdles associated with the practical task of imbalanced medical domain adaptation.

DTTL stands as an illustration of the synergy between advanced machine learning techniques and the foundational concepts of entropy and information theory. By systematically incorporating these elements, the proposed model not only aligns the distributional characteristics of source and target domains but also seeks to correct the imbalances present within class distributions. The emphasis on entropy reduction and the maximization of mutual information across domains highlights our commitment to enhancing the diagnostic model’s efficacy. Through this entropy-informed approach, DTTL endeavors to establish a more robust and reliable framework for medical image diagnosis, particularly in scenarios marred by imbalanced data and domain shifts. Consequently, our work not only contributes to the evolving landscape of medical image analysis but also aligns with the *Entropy* journal’s focus on promoting the integration of entropy and information theory in scientific research.

### 3.1. Overview of Our DTTL Approach

**Figure 2:**
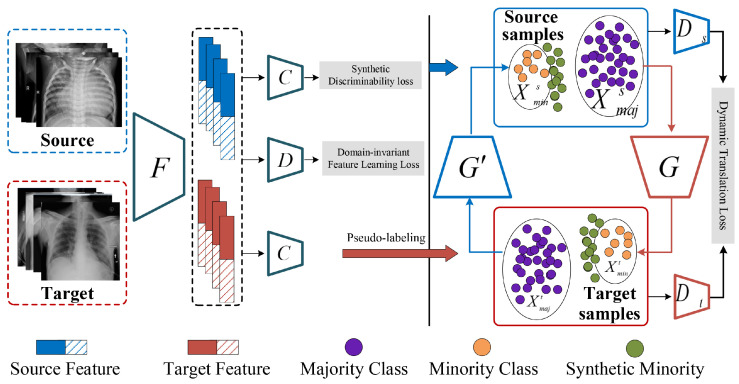
The scheme of the proposed Dynamic Weighting Translation Transfer Learning (DTTL).

The proposed DTTL consists of three key modules, comprising the Cross-domain Discriminability Adaptation (CDA), Dynamic Domain Translation (DDT), and Balanced Target Learning (BTL) units, each incorporating principles of entropy and information theory to enhance the model’s performance in imbalanced medical image classification. To reveal the framework more concisely, we have created the original framework of our model in Figure 2.

First, CDA associates discriminative feature learning between source and target domains, where the synthetic discriminability loss accelerates the discriminative knowledge acquisition for the labeled source and unlabeled target data, and another domain-invariant feature learning loss conducts the knowledge adaptation on the fully unlabeled target domain. This approach utilizes the concept of entropy to maximize the efficiency of knowledge adaptation within the fully unlabeled target domain. With aforementioned pre-trained CDA model, the predictions are transformed into pseudo-labels for all target medical images, which are partitioned into the majority and minority classes ($X_{maj}^{t}$ and $X_{min}^{t}$). Second, the DDT unit develops a dynamic translation process for the imbalanced classes among source and target domains. In detail, it integrates a dynamic transformation loss, inspired by information theory, to map source majority samples into target minority classes based on generative adversarial network, synthesizing minority samples for target domain. This mechanism encourages the translated medical images to be close to the class boundary; a confidence-based selection approach explores the most useful synthesized images to formulate a pseudo-labeled balanced target domain. This unit emphasizes the reduction of prediction entropy, ensuring the generated samples contribute effectively to balancing the target domain. Finally, the Balanced Target Learning module performs supervised training on the reassembled dataset. By applying a training strategy that accounts for entropy minimization in the learning process, this module ensures that the final diagnostic model is robust and capable of handling imbalanced medical image classification tasks with improved accuracy and reliability.

Leveraging these entropy- and information theory-enhanced methodologies, our DTTL framework strives to establish a new standard for imbalanced medical image classification, addressing both domain shift and class imbalance issues more effectively than existing state-of-the-art methods.

### 3.2. Cross-Domain Discriminability Adaptation

In the framework of medical domain adaptation, the source domain with annotations provides essential discriminative features that are crucial for addressing class imbalance in the unlabeled target domain by enriching the target domain’s learning process with relevant information. To achieve this goal, we firstly map source and target samples into a domain-invariant feature space by adversarial learning, which has been successfully involved in domain alignment, inspired by [20,23].

To extract domain-invariant feature representations, we first input source and target medical images into a feature generator *F*, paired with a domain discriminator *D*, both of which parameters $\theta_{f}$ and $\theta_{d}$ are trainable in optimizing the following domain-invariant feature learning (DFL) loss, inspired by GAN [36]:

(1) $$\begin{array}{cc} \min_{\theta_{f}}\max_{\theta_{d}}L_{dfl}\left(\theta_{f},\theta_{d}\right) & =E_{x_{i}^{s}∼D_{s}}\log\left[D\left(F\left(x_{i}^{s}\right)\right)\right]\\ \; & +E_{x_{j}^{t}∼D_{t}}\log\left[1-D\left(F\left(x_{j}^{t}\right)\right)\right] \end{array}$$

The domain-shift problem can be effectively solved by this adversarial learning. Moreover, the preliminary discriminability of the feature generator is optimized by the synthetic discriminability (SD) loss in source and target domains, Equation (2).

(2) $$\begin{array}{cc} \min_{\theta_{f}}L_{sd}\left(\theta_{f}\right)= & -E_{x_{i}^{s}∼D_{s}}y_{i}^{s}\log C\left(F\left(x_{i}^{s}\right)\right)\\ & -\lambda E_{x_{j}^{t}∼D_{t}}C\left(F\left(x_{j}^{t}\right)\right)\log C\left(F\left(x_{j}^{t}\right)\right) \end{array}$$

where *C* is a class prediction layer in source domain and λ is a balance parameter. The above two terms in SD loss are the entropy loss of source samples and the information maximization loss of target data. We propose that the synthetic discriminability loss can align the classification-ability discrepancy between source and target data, and the extracted target feature representations can be highly informative, enabling the classifier *C* to identify target samples.

In an imbalanced medical image classification task, the distribution of majority and minority classes severely interferes with the following training step. Thus, the overall imbalance distribution should be estimated for the unlabeled target domain. Relying on the initially trained feature extractor *F* and classifier *C*, the pseudo-label of each target sample $x_{j}^{t}$ can be inferred by

(3) $$\begin{array}{c} {\hat{y}}_{j}^{t}=\arg\max_{k}C\left(F\left(x_{j}^{t}\right)\right)\left\{k\right\}{)|}_{k\in \left(maj,min\right)} \end{array}$$

where C(·){k} denotes the *k*-th element in the predicted probability vector. After that, the majority and minority classes can be separated from target domain, represented by $X_{maj}^{t}$ and $X_{min}^{t}$, respectively, such that the target dataset is represented by $T=X_{maj}^{t}⋃X_{min}^{t}$.

### 3.3. Cross-Domain Minority Translation

Most medical image diagnosis tasks focus on binary imbalanced classification, such as identification of benign (majority) and malignant (minority) tumors. Inadequate management of imbalanced data distributions can degrade the performance of diagnostic models for minority classes, often biasing the outcome towards excessive classification of the majority class due to its elevated prior probability [37]. Strategies for addressing issues of imbalance in data are primarily divided into two groups: adjusting the loss function’s weighting to emphasize the minority class and altering the dataset through resampling to enhance the visibility of the minority class.

This paper aligns with the second approach, offering a method to augment the target minority class through the creation of synthetic minority instances. These instances are derived from transformations of authentic majority class samples from the source. Given that pseudo-labeled samples from the target majority class inevitably include inaccurately predicted data, utilizing the variance found in actual source majority samples enables this translation method to produce instances that enhance the efficacy of classification.

Inspired by the successful image translation framework CycleGAN [36], it introduces the regularizing loss functions described below that allow us to achieve cross-domain and cross-class image translation task (e.g., source majority sample → target minority samples). We propose a Cross-domain Minority Translation GAN method to translate source majority → target minority and target majority → source minority like a cycle-consistency method based on CycleGAN.

Given the imbalanced source $S=X_{maj}^{s}⋃X_{min}^{s}$ and target $T=X_{maj}^{t}⋃X_{min}^{t}$, we establish two generators, $G:X_{maj}^{s}\to X_{min}^{t}$ and $G^{\prime }:X_{maj}^{t}\to X_{min}^{s}$, with two corresponding discriminators, $D_{s}$ and $D_{t}$, to identify the synthetic minority samples from either the source or target domains, as illustrated in Figure 2 (right).

**Direct translation loss.** To harmonize the central goal of accurately representing the minority distribution with the aim of reducing the discrepancy between the output translated instance and the input instance, we initially present the direct translation (DT) loss alongside the subsequent GAN losses,

(4) $$\begin{array}{c} L_{DT}=E_{x_{j}^{t}∼X_{maj}^{t}}∥G\left(x_{j}^{t}\right)-x_{j}^{t}∥ \end{array}$$

**Minority cycle consistency loss.** The intuitive objective of generators *G* and $G^{\prime }$ is to map the input samples (either the minority or majority) into the cross-domain minority distribution. Thus, a Minority Cycle Consistency (MCC) loss is developed from the cycle-consistency loss in CycleGAN, mathematically formulated as

(5) $$\begin{array}{cc} L_{MCC} & =E_{x_{i}^{s}∼X_{min}^{s}}{∥G^{\prime }\left(G\left(x_{i}^{s}\right)\right)-x_{i}^{s}∥}_{1}\\ & +E_{x_{j}^{t}∼X_{min}^{t}}{∥G\left(G^{\prime }\left(x_{j}^{t}\right)\right)-x_{j}^{t}∥}_{1} \end{array}$$

**Identity loss.** This loss promotes the generators to produce mappings that closely approximate the identity function when inputs are drawn from the target distribution,

(6) $$\begin{array}{cc} L_{IDE} & =E_{x_{i}^{s}∼X_{min}^{s}}{∥G^{\prime }\left(x_{i}^{s}\right)-x_{i}^{s}∥}_{1}\\ & +E_{x_{j}^{t}∼X_{min}^{t}}{∥G\left(x_{j}^{t}\right)-x_{j}^{t}∥}_{1} \end{array}$$

Naturally, these losses motivate the translation process to exhibit certain beneficial characteristics: firstly, that, through reciprocal translation, we aim to restore the input samples to their original state and, secondly, that translation is unnecessary when the data already resides in the target domain.

**Adversarial loss.** Most importantly, the generators *G* and $G^{\prime }$ are responsible for transforming the majority samples into minority across source and target domains, with adversarial learning from discriminators $D_{t}$ and $D_{s}$. The generative adversarial loss is defined by

(7) $$\begin{array}{cc} \; & \min_{D_{s},D_{t}}\max_{G,G^{\prime }}L_{GAN}=L_{GAN}\left(G,D_{t}\right)+L_{GAN}\left(G^{\prime },D_{s}\right)\\ \; & =E_{x_{j}^{t}\in D_{t}}\left[\log D_{t}\left(x_{j}^{t}\right)\right]+E_{x_{i}^{s}\in X_{maj}^{s}}\left[\log\left(1-D_{t}\left(G\left(x_{i}^{s}\right)\right)\right)\right]\\ \; & +E_{x_{i}^{s}\in D_{s}}\left[\log D_{s}\left(x_{i}^{s}\right)\right]+E_{x_{j}^{t}\in X_{maj}^{t}}\left[\log\left(1-D_{s}\left(G^{\prime }\left(x_{j}^{t}\right)\right)\right)\right] \end{array}$$

The final cross-domain minority translation (CMT) objective is given by

(8) $$\begin{array}{c} L_{CMT}=L_{GAN}+\lambda_{DT}L_{DT}+\lambda_{MCC}L_{MCC}+\lambda_{IDE}L_{IDE} \end{array}$$

The cross-domain minority translation workflow is described in Figure 2 and we can obtain sufficient synthetic target minority samples by

(9) $$X_{gen}^{t}=G\left(X_{maj}^{s}\right)$$

### 3.4. Balanced Target Learning

While it is theoretically possible to enrich the dataset with the complete set of $X_{gen}^{t}$, in practice, this collection may include instances that are excessively distant from the class boundary, either too close or too far, potentially causing harm. Specifically, the extra losses introduced by the cross-domain minority translation module prompt the generation of samples nearer to the target class boundary. These samples are more likely to transition into the target majority category. To address this issue, we further proposes a sample selection algorithm for the generated samples $X_{gen}^{t}$, described below.

We reuse the feature generator *F* and classifier *C* in Equation (2), to construct the desired final predictor for the target samples. We employ C(F(·)) to evaluate the probability that each generated target sample in $X_{gen}^{t}$ is part of the target minority class, ranking the samples of $X_{gen}$ by this probability in a descending sequence. A threshold $p_{max}$ is then set to exclude samples with probability scores above this value, which indicates their considerable distance from the class boundary and makes them less beneficial for training. Subsequently, we limit the quantity of chosen samples to a specific multiple *s* relative to the size of the target minority class, to precisely adjust the sample selection.

(10) $$\begin{array}{c} X_{selected}^{t}=sorted\left(\left\{x\in X_{gen}^{t}|C\left(F\left(x\right)\right)\leq p_{max}\right\}\right)\left[:s\right|X_{min}\left|\right] \end{array}$$

Both the threshold probability $p_{max}$ and the retention sample count *s*, defined as a proportion of the minority class size, are regarded as hyperparameters within our model. The augmented and resampled dataset thus generated is represented as

(11) $$\begin{array}{c} T^{\prime }=X_{maj}^{t}\cup {\left\{X_{min}^{t}\cup X_{selected}^{t}\right\}}_{min} \end{array}$$

After augmenting the imbalanced target data, we consider further training the feature generator *F* and classifier *C* by the balanced target samples $x_{i}\in T^{\prime }$ with corresponding labels $y_{i}$ by minimizing the entropy loss defined as following,

(12) $$\begin{array}{c} L_{ent}=-E_{x_{i}\in T^{\prime }}y_{i}\log C\left(F\left(x_{i}\right)\right) \end{array}$$

The overall algorithm is summarized in Algorithm 1.
**Algorithm 1** Training of the DTTL model.**Require:** Source medical images $S=\left\{\left(x_{i}^{s},y_{i}^{s}\right)\right\}{|}_{i=1}^{n_{s}}$ and unlabeled target data $T=\left\{x_{j}^{t}\right\}{|}_{j=1}^{n_{t}}$   
  1:**repeat**    2:    Send the images x∈S to CDA module (Equations (1) and (2)) and predict images into pseudo class-imbalanced labels as $X_{maj}^{t}$ and $X_{min}^{t}$ (Equation (3)).     3:    Translate imbalanced classes between source and target domains by CMT module (Equation (8)) and obtain sufficient synthetic target minority samples (Equation (9)).     4:    Select the synthetic target samples by Equation (10) and constitute the final target domain $T^{\prime }$ (Equation (11)).     5:    Training the target model in the balanced target data $T^{\prime }$(Equation (12)).     6:**until** Convergence;   **Ensure:** The category of target medical images.

## 4. Experiments

### 4.1. Dataset

We assess how effective DTTL is when used for medical image classification on two datasets. Each dataset has been divided into three subsets and Table 1 contains the summarized statistical information. Below are the descriptions of the datasets:

**The Radiological Society of North America (RSNA)** pneumonia detection challenge dataset [38] is divided into two stages, starting with the release of a training set containing 25,684 radiographs and a test set containing 1000 radiographs from the ChestX-ray14 dataset [39]. All radiographs are in an anonymized DICOM format, with a resolution of 1024×1024 pixels and an 8-bit depth. The training images were labeled by non-thoracic radiologists affiliated with RSNA, while the test images were labeled by specialist thoracic radiologists from the Society of Thoracic Radiology. The dataset contains 6011 cases of pneumonia and 20,672 other cases, including normal and other diseases. The distribution of classes is imbalanced, with a ratio of approximately 0.25:1. It is worth noting that our proposed DTTL model is trained using this dataset as the source domain.

The RSNA pneumonia detection challenge dataset boasts a substantial collection of 25,684 radiographs, each meticulously annotated by reputable radiologists, ensuring the precision of the data for robust analysis. As a dataset widely acknowledged in the field of medical image analysis, utilizing it in our experiments provides a credible benchmark against other methods, enhancing the public validation of our approach’s effectiveness. Furthermore, the dataset exhibits an imbalance in disease categories, with a notable predominance of normal cases and other diseases compared to pneumonia cases. This distribution mirrors real-world clinical scenarios, making it an ideal testbed for evaluating our model’s ability to learn from imbalanced data, which is critical for practical applications.

**The Child X-ray** dataset [40] is collected for diagnosing pneumonia in children’s radiographic data. It comprises 5232 chest X-ray images from 5856 patients, including 3883 images showing pneumonia (2538 bacterial and 1345 viral) and 1349 normal cases. To establish an imbalanced class scenario, we selected 300 cases of pneumonia and 1349 normal cases from this dataset as the target domain, aiming to perform transfer learning from the RSNA dataset as the source.

### 4.2. Experimental Settings

Following the standard protocols for unsupervised domain adaptation, all labeled source medical images and unlabeled target samples are incorporated into the model training process. Following traditional settings that learn knowledge from large-scale datasets to adapt to small sets, this paper employs RSNA as the source and the Child X-ray as the target domain to conduct the transfer learning for the imbalanced medical image classification task. In this experiment, the feature extractor *F* utilizes a backbone CNN architecture that includes the widely used convolutional layers from ResNet50 [41], DenseNet121 [42], and EfficientNet-B4 [37]. The cross-domain minority translation module’s GAN framework is adapted from CycleGAN [36]. For optimization, the network weights are trained by Adam with $5\times 10^{-4}$ and the learning rate is set as $2\times 10^{-4}$, with mini-batch size 16. We stop training after the model convergence when the loss variation is lower than $5\times 10^{-4}$ or the training reaches 200 epochs. The classifiers we adopted in the experiment are two-layer network, and the domain discriminator consists of two layers with ReLU and Dropout (0.5) in all the layers. Specifically, we utilize PyTorch to implement our method, and the parameters in loss functions are λ=(Equation (2)), [$\lambda_{DT}=$, $\lambda_{MCC}=$, $\lambda_{IDE}=$] (Equation (8)). The source code will be released in GitHub https://github.com/yucl2019/DTTL (accessed on 23 April 2024).

### 4.3. Compared Methods

In this experiment, we compare DTTL against seven distinct domain adaptation techniques, including Deep Adaptation Neural Network (DANN) [28], Cycle Consistent Adversarial Domain Adaptation (CyCADA) [30], Beyond Sharing Weights (BSW) [31], Conditional Domain Adaptation Network (CDAN) [29], Maximum Classifier Discrepancy (MCD) [32], Margin Disparity Discrepancy (MDD) [33], FixBi [34], Entropy Minimization versus Diversity Maximization (MEDM) [35], and Contrastive Domain Adaptation with Consistency Match (CDACM) [11]. The RSNA and Child X-ray datasets are utilized as the source and target datasets, respectively, and we assess performance using three different backbone architectures: ResNet50 [41], DenseNet121 [42], and EfficientNet-B4 [37].

### 4.4. Results

In the experiments, the dataset of the original Child X-ray dataset [40] was randomly divided into a training set and a testing set, with a ratio of 0.8:0.2 for model training. Table 2 summarizes the statistical results of this method across five runs. As illustrated in the table, the DTTL method significantly outperforms other comparative methods on the Child X-ray dataset, which is clearly reflected from the perspective of AUC scores. Analyzing the data in Table 2, it is evident that the DTTL method achieves the highest AUC scores across three different backbone networks, notably with EfficientNet, DenseNet121, and ResNet50, scoring 91.68%, 90.46%, and 89.90% respectively. These results indicate the DTTL method’s excellent generalization ability and robustness across different network architectures.

Compared to key existing methods, DTTL demonstrates enhanced superior performance across all evaluated backbone architectures. For instance, compared to CDACM, DTTL improved AUC scores by 1.21%, 2.13%, and 1.82% on EfficientNet, DenseNet121, and ResNet50, respectively. As for other methods such as DANN, CyCADA, BSW, CDAN, MCD, MDD, FixBi, and MEDM, DTTL’s advantage is even more pronounced.

These outcomes demonstrate that the DTTL method, regardless of the chosen backbone network, can effectively enhance the classification performance when processing the Child X-ray dataset. This success can be attributed to the DTTL method’s refine optimizations in feature extraction, representation learning, and model generalization, tailored specifically for varied network architectures. Furthermore, the DTTL method exhibits good adaptability to different network structures, maintaining efficient performance across diverse settings.

In summary, the DTTL method showcases significant advantages in the medical image classification realm, particularly evident in its performance on the Child X-ray dataset, and consistently achieves high classification accuracy across a variety of backbone architectures.

To evaluate the algorithm’s robustness in scenarios where the target domain data are limited, the second experiment of this study randomly samples 10% of the data from the training and validation sets previously utilized in the first experiment for model training. Table 3 presents the average statistical outcomes over five runs of this method, facilitating the following conclusions:

The performance of the DTTL method on the Child X-ray dataset, utilizing only 10% of the training and validation data, remains impressive. Within the three backbone networks of EfficientNet, DenseNet121, and ResNet50, the AUC scores for DTTL were 87.23%, 85.28%, and 87.10%, respectively, outperforming other comparative methods in most cases. Specifically, DTTL marginally outperforms CDACM on EfficientNet and DenseNet121, but slightly lags behind ResNet50. Compared to the MDD method, DTTL demonstrates superior performance across all backbone networks, especially on EfficientNet and DenseNet121. These results suggest that DTTL is capable of effectively learning and sustaining robust performance even with scarce training data.

It is noteworthy that, while most methods experienced a decline in performance when the training data was reduced to merely 10%, DTTL exhibited a relatively minor performance drop, further evidencing the robustness of the DTTL method in scenarios with sparse data. For instance, the performance decrease of DTTL on EfficientNet was less pronounced compared to using the full dataset, which may be attributed to its optimized feature extraction and domain adaptation capabilities.

These outcomes highlight the applicability and effectiveness of the DTTL method in medical image classification tasks, particularly when available data is limited. The robustness of the DTTL method suggests that it can still effectively capture and utilize key information to maintain high performance, even with a limited number of training samples.

**Figure 3:**
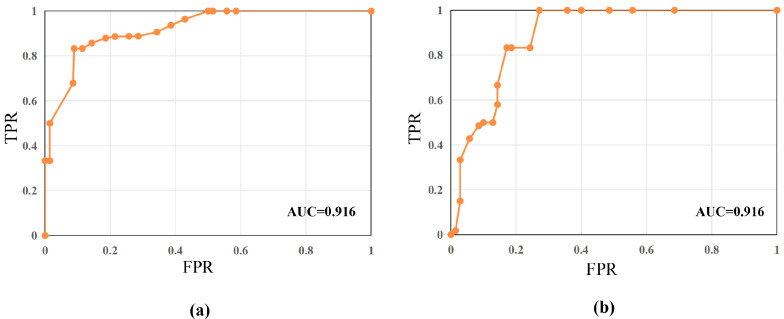
The ROC curve of DTTL model based on EfficientNet on Child X-ray dataset, (**a**) full data and (**b**) 10% data.

To showcase the performance of DTTL further, this section generates a Receiver Operating Characteristic Curve (ROC), demonstrating the model’s classification ability, as shown in Figure 3.

### 4.5. Ablation Study

To further dissect the performance of the Dynamic Weighting Translation Transfer Learning (DTTL) model proposed in this paper, this chapter conducts ablation studies by systematically removing various components of the model, such as the Cross-domain Discriminability Adaptation (CDA) module and the Cross-domain Minority class sample Translation (CMT) module.

#### 4.5.1. Validation of Cross-Domain Discriminability Adaptation

**Figure 4:**
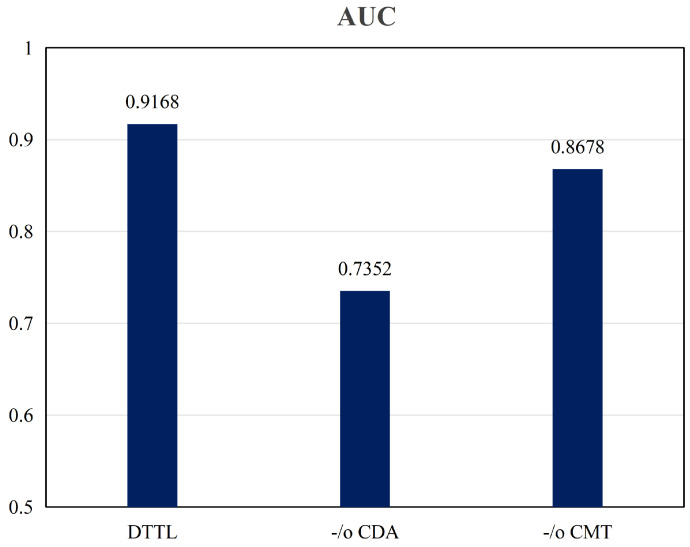
Validation results; -/o CDA represents DTTL removing CDA module; -/o CMT represents DTTL removing CMT module.

This section investigates the impact of the Cross-domain Discriminability Adaptation (CDA) module on the efficacy of the Dynamic Weighting Translation Transfer Learning (DTTL) framework for imbalanced medical image classification tasks. The evaluation concentrates on datasets characterized by substantial domain shifts, such as the RSNA and Child X-ray datasets, representing source and target domains with diverse class distributions. The crux of this study is a comparative analysis of DTTL’s performance with and without the CDA module. The experimental results indicate that, with the incorporation of the CDA module, DTTL achieves an AUC-ROC score of 0.9168, demonstrating strong cross-domain class discriminability. In contrast, DTTL without the CDA module shows a significantly lower AUC-ROC of 0.7352, as depicted in Figure 4. This substantial difference underscores the pivotal role of the CDA module in enhancing the performance of DTTL.

In this part of the experiment, the enhancement in AUC is critical, as the CDA module endeavors to mitigate the domain shift in imbalanced medical image classification. It achieves this by facilitating more discriminative feature learning between the source and target domains. The outcomes of the ablation study presented in this paper substantiate the indispensability of the CDA module within the DTTL framework. By aligning feature representations across domains and promoting domain-invariant feature learning, the CDA module effectively tackles the challenge of domain shift. It aids in transferring the discriminative knowledge from the labeled source domain to the unlabeled target domain, thus enhancing the overall classification performance. The superiority of the DTTL framework with the CDA module is further exemplified by its ability to leverage the discriminative information for better generalization in the target domain, resulting in marked improvements in diagnostic accuracy as reflected in the AUC metric.

#### 4.5.2. Validation of Cross-Domain Minority Class Sample Translation

This section assesses the influence of the Cross-domain Minority class Translation (CMT) module on the efficacy of Dynamic Weighting Translation Transfer Learning (DTTL) framework. The study employs the key performance metric of Area Under the Curve–Receiver Operating Characteristic (AUC-ROC) to measure the contribution of the CMT module to the overall effectiveness. Comparative analysis between DTTL configurations with and without the CMT module indicates a significant performance enhancement when the module is integrated. Notably, the DTTL equipped with the CMT module achieves an AUC-ROC score of 0.9168. In contrast, the AUC-ROC score diminishes to 0.8678 in the absence of the CMT module, as depicted in Figure 4.

These findings highlight the efficacy of the CMT module in addressing the issue of class imbalance in medical image classification. By generating synthetic data that transforms samples of majority classes in the source domain into those of minority classes, the CMT module rebalances the class distribution in the target domain, thereby achieving more precise and reliable classification. Thus, the ablation studies in this part underscore the pivotal role of the CMT module within the DTTL framework. Utilizing generative adversarial networks and a sophisticated translation process, the CMT module creates synthetic samples of minority classes to counteract the imbalance of class distribution. This approach significantly reduces model bias towards majority classes and enhances classification performance for minority classes, attesting to the importance of the CMT module in improving the accuracy and reliability of DTTL in classifying imbalanced medical image datasets.

In our ablation study, the paper examines the contribution of the Cross-domain Discriminability Adaptation (CDA) module and the Cross-domain Minority class Translation (CMT) module to the overall performance of the Dynamic Weighting Translation Transfer Learning (DTTL) framework. The results clearly demonstrate that these two modules play a critical role in addressing the challenges of domain shift and class imbalance in imbalanced medical image classification. By effectively aligning feature representations and adjusting the class distribution, the CDA and CMT modules significantly enhance the accuracy and reliability of the DTTL framework. The findings from this ablation study underline the indispensability of these modules and provide direction for future research in developing more robust transfer learning methods to tackle imbalanced medical image classification challenges.

## 5. Conclusions

In this paper, considering the issues of domain adaptation and class imbalance in medical image classification, a novel approach to Dynamic Weighting Translation Transfer Learning (DTTL) is proposed. This method addresses domain shifts and balances medical image categories across different classes through three pivotal steps, encompassing Cross-domain Discriminability Adaptation (CDA), Dynamic Domain Translation (DDT), and Balanced Target Learning (BTL) units. Each module incorporates entropy and information theory principles to enhance model performance and adaptability.

Specifically, the CDA unit leverages synthetic discriminability feature learning coupled with a domain-invariant feature learning loss. These strategies are grounded in information theory, aiming to minimize the entropy between source and target domain features and maximize the mutual information for effective domain adaptation. The DDT unit implements a dynamic translation process for the imbalanced classes across domains, employing a confidence-based selection method to curate the most useful synthesized images to form a pseudo-labeled balanced target domain, which is instrumental in reducing the overall system entropy and enhancing the reliability of the model’s predictions. Finally, the BTL unit undertakes supervised learning on the reassembled target set. This phase is crucial for refining the model’s ability to classify imbalanced medical images accurately, applying entropy minimization techniques to improve diagnostic performance.

To evaluate the efficacy of DTTL in the classification of imbalanced medical images, modeling experiments were conducted using the RSNA and Child X-ray datasets as the source and target domains, respectively. The evaluation results indicate that the DTTL presented in this paper outperforms existing state-of-the-art methods, providing an innovative solution for transfer learning tasks in the classification of class-imbalanced medical images. This success underscores the potential of incorporating entropy and information theory concepts in developing more effective transfer learning solutions for the classification of class-imbalanced medical images.

Our work not only introduces a novel approach to tackle domain adaptation and class imbalance issues but also emphasizes the significance of entropy and information theory in enhancing the robustness and accuracy of medical image classification models. We believe that DTTL offers a promising direction for future research in transfer learning and medical image analysis.

## Figures and Tables

**Table 1:** The class distribution of each datasets.

Label	RSNA	Child X-ray
Pneumonia	6011	300
Normal and others	20,672	1349

**Table 2:** Performance of comparison of DTTL with the peer methods on Child X-ray in terms of the AUC score (%).

Method	EfficientNet	DenseNet121	ResNet50
DANN [28]	85.42	87.08	86.24
CyCADA [30]	79.76	79.09	77.22
BSW [31]	84.33	86.79	86.37
CDAN [29]	84.33	86.46	86.96
MCD [32]	83.41	85.43	86.51
MDD [33]	88.18	85.11	86.77
FixBi [34]	84.07	87.28	85.88
MEDM [35]	84.65	81.46	82.51
CDACM [11]	90.57	88.33	88.08
DTTL	91.68	90.46	89.90

**Table 3:** Performance of comparison of DTTL with the peer methods on Child X-ray (with 10% training data) in terms of the AUC score (%).

Method	EfficientNet	DenseNet121	ResNet50
DANN [28]	75.87	73.77	73.96
CyCADA [30]	67.71	59.3	68.61
BSW [31]	74.31	54.84	69.99
CDAN [29]	76.25	74.64	75.08
MCD [32]	81.69	81.21	84.72
MDD [33]	84.80	84.23	84.01
FixBi [34]	75.92	73.65	81.14
MEDM [35]	79.86	78.70	70.76
CDACM [11]	86.89	85.04	87.35
DTTL	87.23	85.28	87.10

## Data Availability

The raw data supporting the conclusions of this article will be made available by the authors on request.
